# A cluster randomised, double-blind pilot and feasibility trial of an active behavioural physiotherapy intervention for acute whiplash-associated disorder (WAD)II

**DOI:** 10.1371/journal.pone.0215803

**Published:** 2019-05-09

**Authors:** Taweewat Wiangkham, Joan Duda, M. Sayeed Haque, Jonathan Price, Alison Rushton

**Affiliations:** 1 Centre of Precision Rehabilitation for Spinal Pain (CPR Spine), University of Birmingham, Birmingham, West Midlands, United Kingdom; 2 School of Sport, Exercise and Rehabilitation Sciences, University of Birmingham, Birmingham, West Midlands, United Kingdom; 3 Exercise and Rehabilitation Sciences Research Unit, Naresuan University, Phitsanulok, Thailand; 4 Department of Physical Therapy, Naresuan University, Phitsanulok, Thailand; 5 Institute of Applied Health Research, University of Birmingham, Birmingham, West Midlands, United Kingdom; 6 Physiotherapy Department, Birmingham Community Healthcare NHS Foundation Trust, Birmingham, West Midlands, United Kingdom; Monash University, AUSTRALIA

## Abstract

Whiplash-associated disorder (WAD) causes substantial social and economic burden, with ≥70% patients classified as WADII (neck complaint and musculoskeletal sign(s)). Effective management in the acute stage is required to prevent development of chronicity; an issue for 60% of patients. An *Active Behavioural Physiotherapy Intervention* (ABPI) was developed to address both physical and psychological components of WAD. The ABPI is a novel complex intervention designed through a rigorous sequential multiphase project to prevent transition of acute WAD to chronicity. An external pilot and feasibility cluster randomised double-blind (assessor, participants) parallel two-arm clinical trial was conducted in the UK private sector. The trial compared ABPI versus standard physiotherapy to evaluate trial procedures and feasibility of the ABPI for managing acute WADII in preparation for a future definitive trial. Six private physiotherapy clinics were recruited and cluster randomised using a computer-generated randomisation sequence. Twenty-eight (20 ABPI, 8 standard physiotherapy) participants [median age 38.00 (IQR = 21.50) years] were recruited. Data were analysed descriptively with a priori establishment of success criteria. Ninety-five percent of participants in the ABPI arm fully recovered (Neck Disability Index ≤4, compared to 17% in the standard physiotherapy arm); required fewer treatment sessions; and demonstrated greater improvement in all outcome measures (pain intensity, Cervical Range of Motion, Pressure Pain Threshold, EuroQol-5 Dimensions) except for the Impact of Events Scale and Fear Avoidance Beliefs Questionnaire. The findings support the potential value of the ABPI, and that an adequately powered definitive trial to evaluate effectiveness (clinical, cost) is feasible with minor modifications to procedures.

## Introduction

Whiplash-associated disorders (WAD) describe a range of presentations that may be seen following whiplash injury owing to a wide variety of possible symptoms.[1] WAD is most commonly a consequence of road traffic accidents.[1] WAD is a major public health problem, with high annual economic costs estimated as $242 billion for the USA[2] and €180 billion for Europe.[3] Paralleling increasing healthcare costs are reduced work productivity and earning capacity.[4, 5] For example in Denmark, a decline of 20–25% in employment propensity was observed in the 2 years following a whiplash injury.[4] Insurance companies have also reported an increase in whiplash related costs,[6] particularly in the UK where insurance claim costs are considerable as individuals are largely managed within the private sector by the insurance companies.[6–12] The undesirable title of the ‘whiplash capital of Europe’ has been conferred on the UK by the Association of British Insurers, with estimates that 1 in 140 individuals make claims related to whiplash injury annually.[10] A total of 450,000–580,000 whiplash claims have been reported annually from road traffic accidents in the UK,[13] with estimates for the costs of personal injury claims rising from £7 to £14 billion over a 10 year period.[10] Interestingly, while the costs of whiplash are increasing, the number of patients experiencing WAD is likely to be stable.[13]

These socioeconomic costs are largely explained by up to 60% of individuals with WAD progressing to experience chronic pain and disability; with ~30% people experiencing moderate to severe levels of pain and disability.[14–16] A key consequence of chronicity is a decrease in quality of life for individuals,[17, 18] and a resulting increase in time contributed by caregivers.[4, 5] Unfortunately, approaches to both acute and chronic WAD management have demonstrated limited success.[14, 15, 19–21] Effective management in the acute stage is therefore an important challenge to prevent patients with WAD transitioning to chronicity.[19, 22–24]

The classification of WAD into 5 grades of severity[1] informs clinical reasoning to target interventions to individual patients. The most common classification accounting for ≥70% of individuals is WADII; characterised by a neck complaint accompanied by musculoskeletal sign(s).[25, 26] Individuals with WADII classification are commonly managed by physiotherapists, and in the UK this is usually in the private sector where patients are referred by insurance companies to private physiotherapy clinics.[10] To date, no research has investigated this complex private sector context for managing WAD. Therefore, evaluation of the effectiveness of acute WAD management in the private context is a research priority.

Our recent rigorous systematic review[22, 23] evaluating the effectiveness of acute WADII management found that the combination of active physiotherapy and behavioural interventions termed ‘Active Behavioural Physiotherapy Intervention (ABPI)’, is a potentially effective strategy for the management of acute WADII and may therefore be valuable in preventing chronicity. Unfortunately, the existing evidence was insufficient to define an ABPI intervention. Therefore, an ABPI was developed using empirical and theoretical perspectives through a rigorous process in line with the Medical Research Council Framework of Complex Interventions.[27] An initial modified Delphi study using expertise from international researchers and UK physiotherapists working with WAD patients[28] defined the individual treatment techniques and rehabilitation content of the intervention. Social cognitive theory focusing on self-efficacy enhancement[29] provided the underlying theoretical framework to behaviour change to enable delivery of the intervention. Following development of the ABPI, it was then important to investigate the feasibility of its use in preparation for a future definitive trial. It was important for the research to focus on the private setting in the UK where most patients are managed.[30]

### Aim

To evaluate trial procedures and feasibility of the ABPI in managing acute WADII within the UK insurance private sector to inform the design and sample size requirements for a future definitive RCT.

#### Primary objectives

- To evaluate the feasibility of procedures for a cluster RCT (randomisation, recruitment, data collection, trial management and follow-up)[31–34]
- To evaluate recruitment rates in the private sector in the UK[32, 33]
- To evaluate loss to follow-up of participants in the private sector in the UK[32, 34]

#### Secondary objectives

- To estimate the required sample size for a clustered definitive trial[32–36]
- To evaluate the feasibility of data collection for cost-effectiveness analysis[32]

## Methods

The trial was registered (BioMed Central, ISRCTN84528320) and conducted according to a pre-defined published protocol[37] in order to minimise potential biases, and subsequent deviations were reported. Research methods and reporting were in accordance with the CONSORT 2010 statement: extension to cluster randomised trials.[38] The trial is reported in line with the CONSORT 2010 statement: extension to randomised pilot and feasibility trials[39] (although the CONSORT statement was published after the protocol was developed).

### Trial design

As described in detail previously[37] an external pilot and feasibility trial of a cluster randomised double-blind (assessor, participants), parallel two-arm design, comparing ABPI with standard physiotherapy management, was conducted to evaluate procedures and feasibility of the ABPI (with an embedded qualitative study reported elsewhere). Six private physiotherapy clinics in the West Midlands, UK were recruited. There are many advantages to cluster randomisation in terms of administrative convenience,[40] obtaining the cooperation of investigators, ethical considerations,[40] enhancing participant adherence, reducing treatment contamination,[30, 38, 40, 41] participant blinding,[38] and logistical conveniences.[40] However, the required sample size in a cluster RCT is larger than a parallel design RCT.[42]

In line with the published protocol,[37] 6 private physiotherapy clinics were invited to sign consent forms (cluster-level consent) prior to cluster randomisation.[38] The physiotherapy clinics were randomly allocated to either ABPI or standard physiotherapy by a computer-generated randomisation sequence. Following randomisation, consecutive potential participants referred by an insurance company to the clinics, were screened and recruited by a clinical administrator by telephone to book an initial recruitment appointment. The participant information sheet and consent form were sent via email/post to give potential participants the opportunity to read it in advance of the appointment. During the appointment, the recruiting physiotherapist discussed any issues relating to the trial, afforded an opportunity to ask questions, confirmed eligibility and obtained written consent (individual-level consent). After giving informed written consent, participants were assessed on all outcome measures by a blinded assessor using standardised instruments with established measurement properties. Assessments were made at baseline (following recruitment and consent) and at 3-months post baseline (planned primary endpoint for future definitive trial, the patients with WADII who continue with symptoms and problems after 3 months were defined as chronic).[15, 43] All outcome assessments were independent from treatment sessions and treatment clinics to ensure that the assessor was blinded to treatment allocation. The assessor was a physiotherapist familiar with and trained in use of the outcome measures, and blinded to reduce potential biases. The assessor was not able to access the booking system and participants’ information, while participants did not know to which intervention arm they were allocated, to ensure that both assessor and participants were blinded. To evaluate blinding, at the end of the 3-month follow-up for each participant, the assessor was asked which intervention they thought the patient had received, and the participants were asked which intervention arm they had been allocated to. Two assessment centres central to all clinics enabled convenient attendance for participants. The participants received a text message reminder 2 days prior to the baseline assessment and 3-month follow-up appointments. As part of the consent form, participants were asked to confirm whether they would like their data removed or kept in the trial in the situation that they decided to withdraw (please see online supplementary appendices for the participant information sheet and consent form in the protocol[37]). Participants were invited to provide a reason for their withdrawal.

### Participants

Participants were recruited from the 6 UK private physiotherapy clinics. Demographic characteristics, including age, gender, accident history, present drugs, and information regarding WAD symptoms were collected by the recruiting physiotherapist at the baseline assessment. Participants could claim all expenditure relating their treatment sessions from their insurance company. The trial therefore reimbursed participants for journeys at baseline and 3-month follow-up that were additional contact points.

**Eligibility criteria for clusters**: private physiotherapy clinics in the West Midlands region of the UK. Preliminary data had identified that each clinic had ≥2 patients a month presenting with acute WADII.

**Inclusion criteria**: Participants aged between 18–70 years presenting with WADII[1] from a road traffic accident within the previous 4 weeks.[15, 23, 43–46]

**Exclusion criteria**: Signs and symptoms of upper cervical instability[47] or cervical artery dysfunction,[48] suspected serious spinal pathology, open wounds, active inflammatory arthritis, tumours, infection of the skin and soft tissue, bleeding disorders or using anti-coagulant medication,[47] any current or previous treatment from any other third party, or presenting with any serious injuries from other areas of the body resulting from the accident, history of cervical surgery,[49] previously symptomatic degenerative diseases of the cervical spine within 6 months prior to the road traffic accident,[50] previous history of whiplash or other neck pain,[45] alcohol abuse,[50, 51] dementia,[50, 51] serious mental diseases,[50, 51] psychiatric diseases,[52, 53] and/or non-English speaking and reading. Eligibility criteria were consistent with the published protocol.[37]

### Interventions

Interventions are detailed in line with the Template for Intervention Description and Replication (TIDieR)[54] and trial protocol.[37] Participants in both trial arms attended for face-to-face physiotherapy sessions in a private physiotherapy clinic lasting up to 30 minutes once a week. The total number of treatment sessions varied between 6 to 8, based on the individual physiotherapist’s assessment of the patient’s problems. All physiotherapists in both intervention arms were registered with the UK Health and Care Professions Councils (HCPC), and possessed a minimum of a Bachelor Degree in Physiotherapy and ≥2 years post-registration experience. Fidelity of the ABPI was assessed through the systematic collection of a summary of treatment sessions, and the random observation of sessions by the principal investigator (TW). This enabled monitoring and feedback regarding the intervention to the treating physiotherapist.

#### Standard physiotherapy intervention

Patients were managed according to current practice reflecting the recommendations provided in the clinical whiplash guidelines.[43, 46, 55] Physiotherapy interventions such as reassurance, education, manual therapy, exercise therapy and physical agents, including a home programme of exercises, were part of management depending on the physiotherapist’s decision-making for the individual patient. The treating physiotherapists selected appropriate interventions based on examination findings and clinical reasoning.[48]

#### Active Behavioural Physiotherapy Intervention (ABPI)

The ABPI was developed through the modified Delphi study[28] and social cognitive theory focusing on self-efficacy enhancement.[29] The ABPI consisted of 4 phases in terms of the promotion of understanding, maturity, stamina, and coping.[37] Detail of the ABPI is provided in the published trial protocol.[37] The number of treatment sessions in each phase varied depending on an individual patient’s presentation and problems based on the physiotherapist’s clinical reasoning. Physiotherapists could use a range of techniques (e.g. exercise, relaxation techniques, manual therapy) as part of their ABPI multimodal intervention based on their assessment of an individual patient’s problems using their clinical reasoning. The recommendation was 1–3 visits in each phase.[56]

Physiotherapists were trained to deliver the ABPI in advance of data collection. Training consisted of a group tutorial and workshop followed up with individual training sessions to enable them to tailor the intervention to individual patients with acute WADII based on the findings from the patient history and physical examination data, and their evidence-informed clinical reasoning.[48] The physiotherapists had 4 weeks to practice the skills embedded in the ABPI prior to commencement of participant recruitment. They were randomly observed by TW every week before recruitment commenced and every month during data collection to ensure fidelity of the novel intervention. Feedback was provided throughout the trial.

### Outcomes

As described in detail previously,[37] a range of outcomes were assessed.

#### Primary outcome measure

The Neck Disability Index (NDI) is a patient-reported outcome measure and a valid, reliable and responsive tool for assessing pain and disability of the neck in both acute and chronic conditions.[57–60] It is a self-administered questionnaire consisting of 10 sections focused on pain intensity and functional activities including personal care, lifting, reading, headache, concentration, work, driving, sleeping and recreation.[57] Each section is scored from 0 to 5, with 5 representing the greatest disability. The sum across all sections is calculated to indicate the participant’s self-reported level of disability.[57] The NDI is a robust predictor of outcome for acute WAD[61] and is recommended for monitoring patients with WAD by several clinical guidelines, including the NHS Library, New South Wales Motor Accidents Authority, British Columbia Physiotherapy Association, Royal Dutch Society for Physical Therapy and the South Australian Centre for Trauma and Injury.[43, 46, 59] Consequently, the NDI has been used as the primary outcome in several previous whiplash intervention trials.[20, 21, 45]

### Secondary outcome measures

**Visual Analogue Scale (VAS) for pain intensity**: The most common complaint from patients with WAD is pain.[9] Pain was measured using a Visual Analogue Scale (VAS) from 0mm (no pain) to 100mm (worst possible pain).[62] It is the preferred tool for assessing pain intensity, being simple, and with established high validity and reliability in evaluating acute pain.[63–65] Use of the VAS to identify initial pain intensity has been supported as an important prognostic factor for predicting poor recovery in patients presenting with acute WAD.[61, 66]

**Cervical Range of Motion (CROM)**: Decreased cervical range of motion (CROM) is a common finding in patients presenting with WADII.[67] The measure is sensitive and can discriminate between asymptomatic people and symptomatic whiplash patients,[68] and for handicap prediction from acute whiplash injury.[69] CROM was measured using the cervical range of motion device;[70] a valid and reliable device attached to the head[71–73] while the participant sits on a comfortable chair with both hips and knees flexed to 90°. CROM measurements were recorded 3 times in each direction of motion. The mean of the 3 measurements was used for data analysis.

**Pressure Pain Threshold (PPT)**: Pressure pain threshold (PPT) was measured to identify the threshold of stimulating pain.[74] Patients with WAD frequently reported central hyperexcitability in both acute (≤1 month)[75–77] and chronic presentations.[78] Investigation of PPT at remote pain-free muscle sites provides information on hypersensitivity that may originate from central sensitisation.[79] PPT was measured at symptomatic areas and distal pain-free areas using a digital pressure algometer; a valid and reliable instrument to detect sensitivity.[80, 81] The force was applied at a speed of 30 kPa/s[77] and participants were asked to press a button when their perceived sensation changed from pressure to pain.[77] PPT was assessed at the insertion of the levator scapulae[77] and the upper one-third of the tibialis anterior muscle[81] bilaterally 3 times, with an interval of 1 minute between each measurement.[82, 83] The mean of the 3 measurements was used for data analysis. Positions for testing were comfortable upright sitting with hip and knee flexion to 90° for the levator scapulae, and supine lying with the knee of the assessed side flexed to 90° for the tibialis anterior.

**Impact of Events Scale (IES)**: The Impact of Event Scale (IES) is a 15-item questionnaire assessing current stress and symptoms of post-traumatic stress that may contribute to a high risk of persistent symptoms.[52, 84–86] The IES possesses established reliability and validity,[87–89] and is recommended by guidelines for monitoring whiplash management.[43, 46]

**Fear Avoidance Beliefs Questionnaire (FABQ)**: It is well documented that fear avoidance beliefs and associated behaviours following whiplash injury can influence the physical disability of patients with WAD.[90–92] Patients with any dysfunctional illness beliefs need to have these addressed as part of their management to prevent development of chronicity.[93] The Fear Avoidance Beliefs Questionnaire (FABQ) is a 16-item tool with established reliability and validity for use in populations with neck pain[94]. It is focused to the assessment of a patient’s perceptions of the impact of physical activity and work on their perceived levels of pain and disability[90].

**EuroQol-5 Dimensions (EQ-5D)**: The EQ-5D is a valid and reliable self-report quality of life [95] questionnaire.[96] It is recommended as a useful tool for measuring generic QoL in order to provide information for cost-effectiveness analysis.[97] The EQ-5D has been translated into many languages.[98] In the whiplash literature, the EQ-5D has been used to provide information for cost-effectiveness analysis in one large RCT,[21] directly informing this trial.[37]

### Assessment of outcome

Blinded assessment of outcomes took place at baseline and at 3-months post baseline. After 3 months, the patients with WAD who continued to experience symptoms and problems were defined as chronic.[15, 43] The number of fully recovered patients at 3 months was evaluated. Participants who did not attend the 3-month follow-up assessment were contacted by telephone to make a new appointment. In the situation where participants could not make a new appointment, the assessor asked them to complete the NDI[99] and EQ-5D[100] via telephone interview; both outcomes have established reliability and validity via telephone.

### Feasibility of cost-effectiveness analysis

To assess the feasibility of data collection for the planned cost-effectiveness analysis of a definitive trial, both direct and indirect medical costs were collected. Participants received a diary pocket book to enable recording of any activities related to whiplash management including: medication use, healthcare professional consultations along with any costs they incurred, days off sick, received benefits related to WAD management. General information about participants (including post code, work status and income) was collected on the first page of the diary. Physiotherapy management related costs were collected directly from the physiotherapy clinics. The training costs of physiotherapists in the experimental ABPI trial arm were recorded.

### Sample size

Consistent with this being a pilot and feasibility trial, a power calculation was not required.[32] There is considerable debate around establishing adequate sample sizes for pilot/feasibility trials, and the planned recruitment was for 60 participants (30 in each arm) in order to provide sufficient power of parameters for designing an adequately powered definitive RCT.[101] Physiotherapy clinic data provided evidence of n = 18 eligible participants available each month across the 6 participating clinics. The recruitment rate of the trial was considered adequate if ≥50% of eligible participants were recruited. Based on these estimates, it was anticipated that the trial duration would be 6–7 months for recruitment combined with the 3-month follow-up.

### Randomisation

To minimise the risk of selection bias at the cluster level, Stata software version 12 with blocking, was used to randomise the 6 private physiotherapy clinics into the 2 trial arms: standard physiotherapy intervention (n = 3 clinics) and ABPI (n = 3 clinics). The allocation was concealed prior to assignment, with TW the only investigator involved in the process. Cluster randomisation was implemented in advance of participants being recruited.

### Data analysis

As detailed in the published protocol,[37] data were analysed and summarised using a quantitative synthesis to evaluate eligibility, recruitment and follow-up rates, using IBM SPSS version 22. Consistent with the pilot and feasibility nature of this trial, data were analysed descriptively at the participant level. Descriptive statistics enabled assessment of the feasibility of the ABPI for acute WADII management.[33] Participants who received other treatments from the initial randomised treatment allocation, were retained and their data were included in intention-to-treat analyses. The planned primary endpoint of the future trial is evaluation of the NDI at 3-month follow-up. The intra-cluster correlation coefficient (ICC) was also calculated in order to prepare information for sample size calculation within a clustered definitive trial.[38] The analysis and findings of the quantitative data were discussed by the Acute Whiplash Injury Study (AWIS) steering and data monitoring committee at key stages.

A priori feasibility criteria for progressing to definitive trial were defined (see protocol [37]). Upon completion of the pilot and feasibility trial, the following decisions were possible:

- Stop if the main trial is not possible or valuable
- Continue but modify the protocol if the main trial is possible and valuable
- Continue without modifications but monitor closely if the main trial is possible and valuable with close monitoring
- Continue without modifications if the main trial is possible and valuable.[33]

### Trial management and monitoring

The trial was managed by a Trial Management Group consisting of TW, AR, JD and SH. The Trial Steering Committee and the Data Monitoring Committee functions were combined in line with the trial’s pilot and feasibility nature into the AWIS Steering Group, consisting of TW (principal investigator), AR (chief investigator, lead supervisor, experienced trialist), SH (statistical expert), JP (physiotherapist), a WADII patient, an external member (internationally published whiplash researcher), and an independent chair. The committee met at the start of recruitment, after 3 months of recruitment, and at the completion of data collection. The principal investigator was qualified in Good Clinical Practice [an achievement from the International Conference on Harmonisation of Good Clinical Practice (ICH GCP), certificate number: 33951-36-41796].

### Adverse events

For this trial, adverse events were considered as low risk as WADII is not normally a cause of serious adverse events.[19, 21] In addition, both the ABPI and standard physiotherapy interventions were conservative treatments without existing reporting of serious adverse events.[19, 21] Consequently, patients were unlikely to receive any serious harm from either intervention. In general, only minor adverse events are anticipated after physiotherapy intervention, the most commonly reported being muscle soreness, which usually recovers within 1–2 days.[102]

### Serious adverse events

This trial had a very low risk of serious adverse events in terms of patient pathology, treatment nature and treatment management as only WADII patients were recruited. Participants were evaluated by a physiotherapist prior to seeking consent to ensure that participants were accurately classified as WADII (and so presented only with musculoskeletal signs, with no neurological signs), ensuring that patients with high severity WAD were excluded. In addition, training ensured that all physiotherapists in this trial managed the patients informed by the International Federation of Orthopaedic Manipulative Physical Therapists (IFOMPT) cervical framework,[48] a clinical reasoning framework to identify the risk of adverse events regarding and cervical artery dysfunction of the neck. The definition of a serious adverse event was worsening symptoms within 3 days and being admitted to the hospital due to whiplash problems. In the event of a serious adverse event occurring, participants were able to continue with the trial when their symptoms were resolved.

### Procedures for reporting adverse and serious adverse events

All clinics were provided with an adverse event reporting form. If a participant experienced any unpleasant symptoms, they were asked to report them to their treating physiotherapist. Physiotherapists were required to report any event to TW within 24 hours, and TW was required to report to the AWIS steering committee within 24 hours. This enabled prompt analysis of the event and decision-making regarding any required action. Although not anticipated, any unexpected serious adverse events were required to be immediately reported with an immediate written form and verbal contact by the physiotherapist to TW. Subsequently, TW would report any event to the AWIS steering committee immediately.

### Research governance

The trial maintained research governance by using the principles of the Research Governance Framework for Health and Social Care, in line with University procedures.

### Data management

All information collected about and from the participants was kept safely from any third party to maintain participants’ privacy. All collected documents were stored in a secure place at the School of Sport, Exercise and Rehabilitation Sciences, University of Birmingham. All electronic data were confidentially stored in a password-protected computer. Data could only be accessed by leading members of the research team. All data will be securely destroyed after 10 years.

### Ethical and R&D considerations

NHS ethical approval and R&D approval were not required as the trial sites were in the private setting, outside of the UK National Health Service. The insurance/private clinics did not require further regulatory approval. Written support for the trial was put in place by the private clinics and the insurance companies. Ethical approval was provided by the University of Birmingham Research Ethics Committee following their detailed review *(ERN_15–0542)*.

## Results

### Participant recruitment

Twenty-eight patients were recruited between 06/11/2015 and 01/07/2016 and were followed up for a 3-month period. The trial was stopped early by consensus from the AWIS Steering Group owing to timescale, budget and a reduction of the number of referrals. Two hundred and forty (136 in the ABPI arm and 104 in the standard physiotherapy arm) potential participants were assessed for eligibility by the administrators. Table 1 provides the issues affecting participants’ decisions to not participate in this trial and the administrator’s decision. Reasons for patients’ ineligibility included: ‘post four weeks after road traffic accident’, ‘serious symptom(s) in other regions of the body besides the neck’, ‘having treatment with another clinic’, ‘history of cervical surgery’ and ‘non-English speaking’. Reasons for potential participants declining included: ‘did not want to participate’ and ‘work commitment’. Other reasons included: ‘unable to book initial assessment within four weeks’ and ‘did not want to travel to assessment centre (different physiotherapy clinic)’.

**Table 1:** Issues affecting participants’ decision to not participate (based on administration data).

Category of reasons	ABPI (n = 109)	Standard PT (n = 91)
Reasons for ineligibility (obtained from clinical admin team)		
➢ Post four weeks after road traffic accident	54	42
➢ Serious symptoms in other regions	10	6
➢ Having treatment with another clinic	8	4
➢ History of cervical surgery	3	2
➢ Non-English speaking	1	-
Reasons for declining (obtained from patients by clinical admin team)		
➢ Did not want to participate	10	12
➢ Work commitments	19	22
Other reasons		
➢ Unable to book initial assessment within four weeks	2	1
➢ Did not want to travel to assessment centre (different physiotherapy clinic)	2	2

ABPI, active behavioural physiotherapy intervention; PT, physiotherapy.

**Fig 1:**
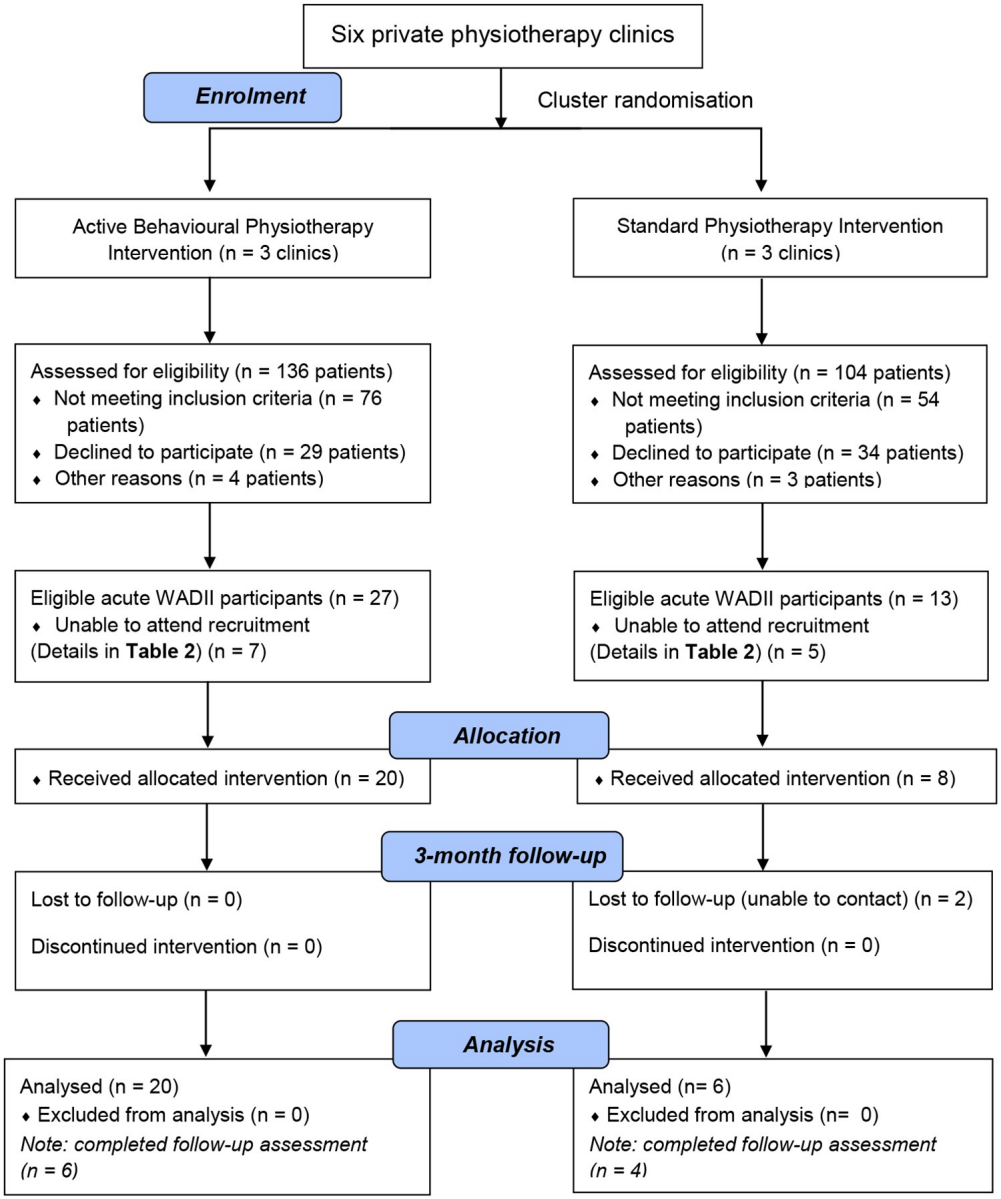
CONSORT flow diagram (adapted from CONSORT 2010).

Twenty-seven in the ABPI arm and 13 in the standard physiotherapy arm eligible participants were booked to attend the initial assessment to confirm eligibility, provide consent and enable baseline assessment data to be collected. Seven eligible participants from the ABPI arm and 5 from the standard physiotherapy arm could subsequently not attend this initial appointment. Their reasons are provided in Table 2. Therefore, 28 out of 40 eligible patients with acute WADII gave their consent and were entered into the trial (20/27 (74.07%) in the ABPI arm and 8/13 (61.54%) in the standard physiotherapy arm). The CONSORT diagram (Fig 1) presents participant progression through the trial.

**Table 2:** Eligible patients interested in participating but unable to attend recruitment.

Category of reasons	ABPI arm (n = 7)			Standard physiotherapy arm (n = 5)		
	WB	ML	SH	GB	BC	SC
Travel issues to assessment centres	3	-	-	1	1	-
Work commitment	-	1	2	-	-	2
Booking patients would like to reschedule but unable to book an initial assessment within 4-week post injury	-	-	1	-	-	1

ABPI, active behavioural physiotherapy intervention; WB, West Bromwich; ML, Moseley; SH, Solihull; GB, Great Barr; BC, Birmingham City; SC, Sutton Coldfield.

### Baseline data

#### Characteristics of participants by intervention arm

The median age of participants was 38.00 (range 22 to 70, IQR: 21.50) years. Table 3 presents the participants’ characteristics by intervention arm at baseline. The median ages of participants in the ABPI and standard physiotherapy arm were 34.00 (IQR = 16.00, range: 22 to70) and 50.50 (IQR = 18.75, range: 26 to 70), respectively. More males were recruited to the ABPI arm than females (17:3), whereas there were more females than males in the standard physiotherapy arm (2:6). White British was the most common ethnic group represented in both arms.

**Table 3:** Participants’ characteristics by intervention arm at baseline.

Demographic category	ABPI (n = 20)	Standard physiotherapy (n = 8)
Age (range, median (IQR))	22 to 70, 34.00 (16.00)	26 to 70, 50.50 (18.75)
Gender (male:female)	17:3	2:6
Ethnic group	White (n = 9)	White (n = 6)
	Asian (n = 7)	Asian (n = 1)
	Chinese or other (n = 2)	Chinese or other (n = 1)
	Black (n = 1)	
	Mixed (n = 1)	

ABPI, active behavioural physiotherapy intervention.

#### Characteristics of physiotherapists by intervention arm

Table 4 presents characteristics of physiotherapists by intervention arm. The median ages of physiotherapists in the ABPI and standard physiotherapy arms were 27 (IQR = 0, range: 23 to 31) and 28 (IQR = 0, range: 26 to 30) years, respectively. All physiotherapists in the ABPI arm were male. Two Britons qualified with bachelor degrees in physiotherapy and one Greek qualified with a master degree in advanced musculoskeletal physiotherapy delivered the ABPI. One Briton (male) and one Greek (female) qualified with bachelor degrees in physiotherapy and one Greek (male) qualified with a master degree in advanced musculoskeletal physiotherapy delivered the standard physiotherapy. The physiotherapists’ duration of experience post qualification was the same in both arms, with a median of three (IQR = 0, range of the ABPI arm: 2 to 4, range of the standard physiotherapy: 2 to 6) years.

**Table 4:** Characteristics of physiotherapists by intervention arm.

Categories	ABPI (n = 3)	Standard physiotherapy (n = 3)
Age (years)		
Median (IQR)	27.00 (0.00)	28.00 (0.00)
Range	23 to 31	26 to 30
Gender (male:female)	3:0	2:1
Ethnicity (n)	British (2) Greek (1)	British (1) Greek (2)
Physiotherapy qualification (n)	Bachelor (2) Master (1)	Bachelor (2) Master (1)
Physiotherapy years of experience		
Median (IQR)	3.00 (0.00)	3.00 (0.00)
Range	2 to 4	2 to 6

ABPI, active behavioural physiotherapy intervention; IQR, interquartile range.

#### Numbers analysed

For each group, all participants were analysed based on their original assigned intervention arms (Fig 1: CONSORT diagram).

### Outcomes and estimation

#### Primary and secondary outcome measures

Primary and secondary outcome measures at baseline and 3 months are descriptively presented in Tables 5 and 6 (baseline scores of participants followed up and lost to follow-up). At 3 months, scores on the NDI, VAS (pain intensity), IES, FABQ, and EQ-5D total and subscales were reduced in both trial arms. The only exception was the usual activities subscale of the EQ-5D, where no difference was observed between baseline and 3-month follow-up scores in the standard physiotherapy arm. The EQ-5D VAS scores in both trial arms were improved at 3 months compared with baseline. Similarly, physical assessments (all planes of CROM and PPT of the levator scapulae and tibialis anterior muscles) were improved in both intervention arms.

**Table 5:** Primary and secondary outcome measures at baseline and three-month follow-up.

Outcome measures	ABPI			Standard physiotherapy		
	Baseline (n = 20) Median (IQR)	3-month Median (IQR)	Median of difference (IQR)	Baseline (n = 8) Median (IQR)	3-month Median (IQR)	Median of difference (IQR)
NDI	17.50 (18.00)	1.00 (2.75) *n = 20*	16.50 (17.25)	21.50 (15.50)	8.00 (8.75) *n = 6*	6.50 (12.50)
VAS	55.50 (29.50)	3.50 (8.25) *n = 6*	48.50 (37.25)	47.00 (31.25)	14.50 (14.75) *n = 4*	37.00 (49.75)
IES	29.50 (31.75)	7.50 (30.50) *n = 6*	13.50 (22.00)	48.00 (32.25)	26.00 (49.75) *n = 4*	24.00 (36.50)
FABQ	60.00 (25.00)	38.00 (19.24) *n = 6*	9.50 (33.00)	61.50 (22.25)	25.50 (19.75) *n = 4*	22.00 (31.00)
EQ-5D Total	11.00 (5.50)	6.00 (1.75) *n = 20*	5.50 (4.75)	10.50 (7.00)	8.50 (4.50) *n = 6*	2.00 (3.00)
EQ-5D Mobility	2.00 (2.00)	1.00 (0.00) *n = 20*	1.00 (1.75)	2.50 (1.75)	1.00 (1.25) *n = 6*	0.50 (1.25)
EQ-5D Self-care	2.00 (1.75)	1.00 (0.00) *n = 20*	1.00 (1.00)	2.00 (0.75)	1.00 (1.00) *n = 6*	0.00 (0.25)
EQ-5D Usual activities	3.00 (1.75)	1.00 (0.00) *n = 20*	1.50 (1.00)	2.00 (1.00)	2.00 (0.25) *n = 6*	0.00 (0.25)
EQ-5D Pain/discomfort	3.00 (0.75)	1.00 (1.00) *n = 20*	2.00 (1.00)	3.00 (1.75)	2.00 (0.50) *n = 6*	0.00 (1.00)
EQ-5D Anxiety/depression	2.00 (2.00)	1.00 (0.00) *n = 20*	1.00 (1.00)	2.50 (1.00)	1.50 (2.50) *n = 6*	0.00 (2.25)
EQ-5D VAS	57.50 (32.50)	98.50 (8.00) *n = 20*	27.00 (24.75)	67.50 (45.50)	75.50 (34.75) *n = 6*	-2.00 (19.00)
CROM Flexion	22.50 (7.67)	46.50 (15.50) *n = 6*	27.67 (14.00)	29.00 (13.24)	47.00 (25.34) *n = 4*	17.34 (21.01)
CROM Extension	22.83 (17.58)	36.50 (30.50) *n = 6*	21.17 (23.59)	19.83 (24.83)	46.33 (24.50) *n = 4*	14.83 (35.33)
CROM Left rotation	29.67 (18.33)	54.00 (16.08) *n = 6*	22.00 (28.42)	40.67 (25.01)	49.67 (24.50) *n = 4*	-1.00 (26.00)
CROM Right rotation	30.67 (17.83)	53.34 (25.17) *n = 6*	32.00 (26.91)	36.34 (22.16)	45.00 (17.34) *n = 4*	4.34 (12.00)
CROM Left lateral flexion	22.34 (13.33)	34.17 (8.67) *n = 6*	11.50 (16.58)	26.00 (12.83)	26.67 (12.67) *n = 4*	1.17 (13.25)
CROM Right lateral flexion	22.67 (11.84)	36.50 (10.75) *n = 6*	11.17 (15.50)	22.17 (10.84)	29.34 (8.42) *n = 4*	6.67 (8.08)
PPT Left levator scapulae	74.67 (71.75)	168.67 (180.66) *n = 6*	90.33 (110.99)	58.67 (36.66)	109.34 (71.08) *n = 4*	63.67 (79.67)
PPT Right levator scapulae	71.50 (69.66)	197.17 (157.50) *n = 6*	121.50 (118.33)	77.17 (44.00)	134.00 (67.59) *n = 4*	49.67 (83.09)
PPT Left tibialis anterior	106.17 (101.08)	223.17 (228.33) *n = 6*	49.84 (129.75)	103.17 (41.08)	168.00 (233.42) *n = 4*	72.67 (192.42)
PPT Right tibialis anterior	90.17 (110.34)	211.84 (233.50) *n = 6*	101.01 (105.00)	88.50 (24.51)	163.67 (181.91) *n = 4*	86.00 (160.58)

ABPI, Active Behavioural Physiotherapy Intervention; NDI, Neck Disability Index; VAS, Visual Analogue Scale; IES, Impact of Events Scale; FABQ, Fear Avoidance Belief Questionnaire; EQ-5D, EuroQol-5 Dimensions; CROM, cervical range of motion; PPT, Pressure Pain Threshold.

**Table 6:** Secondary outcome measures at baseline of followed up and lost to follow-up participants.

Outcome measures	ABPI		Standard physiotherapy	
	Followed up (n = 6) Median (IQR)	Lost to follow-up (n = 14) Median (IQR)	Followed up (n = 4) Median (IQR)	Lost to follow-up (n = 4) Median (IQR)
VAS	58.00 (33.00)	52.50 (32.50)	54.50 (48.00)	46.00 (46.25)
IES	25.50 (26.25)	37.00 (33.50)	50.00 (13.25)	33.50 (41.25)
FABQ	53.00 (30.00)	62.50 (23.25)	59.00 (29.75)	61.50 (20.00)
CROM Flexion	22.33 (9.26)	23.34 (8.92)	26.17 (13.67)	29.17 (20.83)
CROM Extension	18.00 (29.58)	23.33 (12.67)	28.83 (22.51)	12.67 (24.75)
CROM Left rotation	29.00 (21.34)	29.67 (18.83)	45.34 (11.17)	24.67 (25.17)
CROM Right rotation	16.17 (24.67)	31.84 (14.83)	41.00 (22.67)	25.00 (23.50)
CROM Left lateral flexion	21.00 (20.17)	22.34 (11.67)	26.67 (3.41)	19.17 (21.00)
CROM Right lateral flexion	20.33 (21.75)	22.67 (9.83)	22.17 (16.00)	22.34 (14.42)
PPT Left levator scapulae	75.00 (121.84)	74.67 (68.16)	58.50 (32.92)	66.67 (64.08)
PPT Right levator scapulae	69.84 (204.92)	71.50 (53.66)	79.83 (33.00)	60.17 (61.17)
PPT Left tibialis anterior	124.67 (128.42)	99.67 (95.33)	102.67 (61.33)	110.67 (40.83)
PPT Right tibialis anterior	110.67 (157.58)	86.17 (83.83)	81.50 (25.34)	97.17 (31.50)

ABPI, Active Behavioural Physiotherapy Intervention; VAS, Visual Analogue Scale; IES, Impact of Events Scale; FABQ, Fear Avoidance Belief Questionnaire; CROM, cervical range of motion; PPT, Pressure Pain Threshold.

At the 3-month follow-up by intervention arm, the NDI, VAS (pain intensity), IES, EQ-5D (total and all subscales) were reduced in the ABPI arm more than in the standard physiotherapy arm. However, the standard physiotherapy arm had a lower score in the FABQ than in the ABPI. The scores of EQ-5D VAS and physical assessments in the ABPI arm were improved more than the standard physiotherapy arm, with the exception of sagittal cervical movement.

The median of difference in each outcome measure is descriptively provided in Table 5. The NDI, VAS (pain intensity) and EQ-5D total and all subscales in the ABPI arm were reduced more than the standard physiotherapy arm. Moreover, the EQ-5D VAS, CROM all directions and PPT bilaterally for both the levator scapulae and tibialis anterior muscles (except for the left tibialis anterior muscle, which exhibited greater improvement in the standard physiotherapy arm than in the ABPI arm) improved more in the ABPI arm when contrasted to the standard physiotherapy arm. However, the psychological outcome measures (IES and FABQ) were improved more in the standard physiotherapy arm than in the ABPI arm.

At the 3-month post baseline follow-up, 19/20 (95%) participants in the ABPI arm were fully recovered (NDI ≤4). In the standard physiotherapy arm, 1/6 participants (~17%) was fully recovered. Within the subgroup of participants who provided face-to-face assessment at the 3-month follow-up, 5/6 (~83%) participants in the ABPI arm were fully recovered. In the standard physiotherapy, no (0/4) participants were fully recovered. Table 6 enables a comparison of baseline data for participants followed up versus those lost to follow-ups.

#### Information regarding cost-effectiveness

Table 7 provides information about the cost-effectiveness of the 2 treatment arms, and illustrates that the number of treatment sessions and physiotherapy management costs in the ABPI arm were lower than in the standard physiotherapy arm. However, the physiotherapists in the ABPI were trained to deliver the intervention, which cost approximately £200.

**Table 7:** Cost-effectiveness information.

Categories	ABPI (n = 20)	Standard physiotherapy (n = 8)
Treatment sessions (median, IQR)	4.00 (4.00)	6.00 (4.50)
Physiotherapy costs (median, IQR)	£ 90.00 (70.00)	£ 120.00 (75.00)
Physiotherapists’ training costs	£200	-

ABPI, active behavioural physiotherapy intervention.

Only 2 participants in the ABPI arm and none of the participants in the standard physiotherapy arm returned their diary pocket book, and so the data are not reported.

#### Coefficient of intracluster correlation (ICC) and sample size calculation for a cluster RCT

The ICC was calculated using the NDI (primary outcome measure) to inform the design effect or inflation factor[103] prior to calculation of the sample size for a cluster RCT. Based on the findings of this pilot and feasibility trial (variance between clusters = 16.574, variance within clusters = 25.367 + 3.116 = 28.483), ICC = 0.368, Design Effect or inflation factor = 4.312 (using cluster size = 10), the required sample size for a definitive RCT is 22 patients per arm based on power = 90%, significance level = 0.05 and difference of NDI = 8 based on minimal clinically important difference.[104] Consequently, the sample size under cluster RCT is therefore ~190 patients. The required sample size for a definitive cluster RCT is 238 patients based on an estimation of loss to follow-up of 20%. Thus, the required number of clusters is ~24 physiotherapy clinics based on the cluster size = 10.

#### Serious adverse events

No serious adverse event was reported in this trial.

#### Blinding evaluation

The views of both participants and assessor were evaluated at 3-month follow-up with regards to the effectiveness of blinding of this trial by TW. All participants who attended the face-to-face 3-month follow-up (n = 10; 6 from ABPI and 4 from standard physiotherapy arms) and the assessor replied ‘don’t know’ to this question.

## Discussion

### Participant recruitment

There were several factors for not reaching the targeted sample size although the trial recruitment period was extended to July 2016 (should have finished by May 2016 based on the early feasibility data in the protocol[37]) under the oversight of the AWIS Steering Group. The first factor was the unexpected liquidation of the private physiotherapy company initially involved in this trial. Consequently, the trial was temporarily halted from 12/12/2015 to 13/03/2016. Fortunately, an insurance company took over the private physiotherapy company and after considerable negotiation agreed to continue the trial. To ensure the fidelity of the ABPI delivery after the temporary break in the trial, all physiotherapists in the ABPI arm were individually retrained. Secondly, a key reason for potential participants not participating was that they did not want to travel to a different physiotherapy clinic for the assessments (2 options for clinics). Furthermore, although the closing time of one assessment centre was 9.00 pm on Fridays and another centre provided service on Saturdays to be flexible around work commitments, several potential participants declined due to their own work-related constraints. Thirdly, another key consideration that affected recruitment was the takeover of the clinics by one insurance company, as this meant that the other insurance companies did not want to continue to refer their clients. These issues illustrate the complexities of research in the private sector.

According to the CONSORT diagram (Fig 1), the substantial difference in the number of participants between the intervention arms was caused by both an inequality in the number of referrals and the declining of potential participants. In this trial, two levels of randomisation were implemented to minimise the unanticipated difference of the number of referrals between the intervention arms. Randomisation attempted to compromise the difference of the number of participants between intervention arms using 2-level cluster randomisation (large-size physiotherapy clinics were randomly divided into 2 groups first and then the smaller clinics were randomly allocated based on provided information). Unfortunately, the numbers of eligible and recruited participants between the intervention arms were still substantially different.

### Characteristics of participants and physiotherapists

The median age of participants in each intervention arm was substantially different (34 (IQR = 16.00) years in the ABPI and 50.50 (IQR = 18.75) years in the standard physiotherapy). This may have been a factor that explained the differences seen descriptively in recovery between the 2 arms (ABPI 19/20 = 95%; standard physiotherapy 1/6 = 16.7%). However, one meta-analysis of prognostic factors for persistent WAD compared older and younger participants and found that older age (≥50–55 years old) was not a significant factor (OR = 1.00, 95%CI: 0.97 to 1.04) for the risk of persistent pain and disability.[61] Additionally, the proportion of males and females was different across the 2 arms (there were more males than females in the ABPI and vice versa in the standard physiotherapy). The influence of gender is supported by meta-analysis data,[61] which found that females tended to have significantly more persistent problems than males (OR = 1.64, 95%CI: 1.27 to 2.12). However, analysis of the odds ratio suggests that the difference in the proportions of participants with persistent symptoms between genders was low.[61] One cross-sectional study found that the duration of work experience post qualification and level of qualification positively correlated with the level of knowledge in managing musculoskeletal conditions.[105] In this trial, the characteristics of physiotherapists in both arms were similar, giving confidence in findings.

### Outcomes and estimation

In accordance with its pilot and feasibility nature,[33] the results were descriptively reported. Key findings from this trial support that the ABPI may be an effective intervention in managing patients with acute WADII to prevent chronicity. Specifically: 1] Participants in the ABPI arm experienced improved recovery compared to the standard physiotherapy arm in most outcome measures. The exception was the IES and FABQ, but owing to the substantial difference of the number of participants between the intervention arms and the small total sample size, this needs to be investigated further; 2] The median of difference of the planned primary outcome measure (NDI) between baseline and 3-month follow-up reached the minimal clinically important difference in the ABPI arm (NDI ≥8),[106] whereas in the standard physiotherapy arm it did not; 3] The number of fully recovered participants at 3-month follow-up was 19/20 (95%) in the ABPI arm and 1/6 (~17%) in the standard physiotherapy arm when considering a cut off of NDI ≤4.[15, 57, 60, 107].

The loss to follow-up on the primary outcome in this trial was low owing to telephone follow-up strategy, although the majority of participants were young males who tended to drop out more than older males and females.[108] In the ABPI arm, there was no loss to follow-up whereas 2 (25%) participants in the standard physiotherapy arm were lost to follow-up. The low loss to follow up of ~7% at 3-months was less than previous trials (>16% at 6-week follow-up).[47, 109] A useful strategy for ensuring low loss to follow-up was telephone follow-up, which is valid and reliable.[99] However, a key limitation of using telephone follow-up was the lack of physical assessments and the complete range of self-reported outcome measures (owing to feasibility, validity and reliability for the assessment via telephone). In regard to the evaluation of pain intensity via telephone in future research, the numerical rating scale (NRS) (more valid verbal assessment of pain intensity via telephone than VAS) should be used as an outcome measure rather than the VAS.[110]

### Strengths

This trial is the first investigating WAD management in the UK private insurance setting. The ABPI is a novel potentially effective intervention for the management of acute WADII bearing in mind the number of fully recovered participants (NDI ≤ 4)[15, 57, 60, 107] at 3-month follow-up. The ABPI could contribute to reducing the costs of WAD management (lower number of treatment sessions and reduced costs of physiotherapy management than standard physiotherapy). The findings of this trial can be considered reliable due to the high quality of the methodology used in terms of:

- Conducting and reporting in accordance with the CONSORT 2010 statement: extension to cluster randomised trial[38] and also reporting in line with the CONSORT 2010 statement: extension to randomised pilot and feasibility trials.[39]
- A cluster RCT to avoid treatment contamination, increasing participant adherence,[30, 38] participant blinding,[38] and logical and administrative convenience.[40]
- An effective double-blind design to reduce risk of bias.
- Using and training an independent assessor in all outcome measures prior to conducting the trial, leading to reliable results.
- Precision and fidelity in delivering the ABPI to physiotherapy practice (e.g. setting one training day and four weeks for the individual training, systematic treatment recording and random observation of physiotherapists in the ABPI arm every month).

### Limitations

This trial was stopped by the consensus of the AWIS Steering Group (due to timescale constraints, budget and low number of referrals), even though the trial did not reach the target sample size predominantly due to the unexpected liquidation of the private physiotherapy company. Moreover, data regarding level of education (less than post-secondary), reported headache at inception and low back pain at baseline were not collected from the participants, and have now been identified as significant predictors for persistent WAD [61]. The diary pocket book did not work with regard to collecting information for a cost-effectiveness analysis and requires review. The high loss to follow-up for secondary outcome measures is a key limitation, although Table 6 comparing baseline data for participants followed up versus those lost to follow-up does not demonstrate any consistent trends. Finally, the small sample size in the control group and the large disparities in age and gender of the participants between groups are key limitations.

### Considerations for a future definitive trial

Table 8 details the a priori criteria for consideration for a future definitive trial.[37] An adequately powered cluster RCT was deemed feasible with minor modifications.

**Table 8:** Considerations for a future definitive trial.

Objectives	Criteria for success	Considerations
To evaluate the feasibility of procedures (e.g. randomisation, recruitment, collecting data, management and follow-up)	The trial would be considered feasible if it was run smoothly without serious problems or obstructions that were able to stop the study.	All research procedures were feasible but the following issues should be considered:
∘ Randomisation		➢ No issue regarding the randomisation (i.e. no report regarding participants’ disagreement with treatment allocation).
∘ Recruitment		➢ Ideally, double blinding should be kept in order to maintain the quality of the trial but more assessors need to be provided for every clinic in order to reduce the risk factor of journey issues (patients did not want to travel to other physiotherapy clinics) if a future trial is to be sufficiently funded.
		➢ Increase the number of recruited physiotherapy clinics/insurance companies in order to increase the recruitment rate.
		➢ An increase in the number of assessors may be considered. Setting assessment centres did not work in this trial due to participants’ journey issues. It would be ideal to have an assessor in each clinic to enable the baseline assessment to take place local to each clinic prior to the first treatment session. That would then stop the patient needing to make the separate journey for the assessment or travelling to different physiotherapy clinics.
∘ Collecting data		➢ Information for cost-effectiveness analysis should be considered in another way (set up an electronic system by collaborating with an insurance company or a physiotherapy company in order to record relevant information rather than giving a diary pocket book to participants).
		➢ Collecting level of education (less than post-secondary), headache at inception and low back pain, which are the significant predictors of persistent WAD.
∘ Management		➢ No difficulty with the management for the trial.
∘ Follow-up		➢ Face-to-face follow-up may be an issue because participants get back to their normal life and they may not want to come to a clinic owing to their work commitments. Telephone follow-up may be an interesting option for a future trial.
To evaluate recruitment rates, refusal rates and retention in the private sector in the UK	The trial would be considered feasible if ≥ 50% of eligible patients were recruited At least 3 participants a week per intervention arm were recruited ≥ 80% of all recruited participants completed the follow-up at 3 months	Overall, the trial was feasible as: 70% of eligible patients were recruited An average of one (1.27) person was recruited per week (excluding temporary stopping of the trial). This point was an issue to modify in the future trial. An increase in the number of recruited physiotherapy clinics may be an option. ~93% of recruited participants completed 3-month follow-up
To evaluate dropout rates of participants in the private sector in the UK	The trial would be considered feasible if ≤ 20% of all recruited participants dropped out	2/8 (25%) participants were lost to follow-up at 3 months. Therefore, the overall dropout in this trial was ~7%.
To estimate the required sample for a definitive trial	The trial would be considered feasible if it was feasible to achieve the sample size for a cluster RCT based upon recruitment data	The required sample size for a cluster RCT is 238 patients using 24 physiotherapy clinics based on power = 90%, significance level = 0.05, difference of NDI = 4 and cluster size = 10.
To evaluate the feasibility of data collection for cost-effectiveness analysis	The trial would be considered feasible if the following components of the cost-effective analysis were collected with minimal missing data: General information (e.g. current work status and salary) Direct medical costs Medical costs (e.g. physiotherapy, general practice and complementary medicine) Resource uses (e.g. diagnosis tests) Indirect medical costs Participant journey costs Training costs for physiotherapists in the experimental arm	Only 2 participants returned their diary pocket book. Another strategy for collecting information for cost-effectiveness analysis should be considered in another way for a future trial. Setting up an electronic recording system by collaborating with an insurance company or a physiotherapy company may be a good option in order to collect relevant information.

WAD, whiplash-associated disorder; RCT, randomised controlled trial; NDI, neck disability index.

## Conclusion

This is the first trial investigating WAD management in the UK private insurance setting, and highlights the challenges for future research. The findings suggest that the ABPI is feasible (with regard to procedures, sample size and modified collection of data for cost-effectiveness analysis) and valuable (higher proportion of completely recovered participants, fewer treatment sessions, and reduced physiotherapy management costs than the standard physiotherapy). The findings support the appropriateness of conducting a future definitive trial to evaluate the effectiveness of the ABPI for the management of acute WADII with minor modifications.

## Supporting information

S1 Checklist CONSORT 2010 checklist. (DOC)

S1 Data Full data set. (SAV)
